# Prostate-Derived ETS Factor (PDEF) Modulates Yes Associated Protein 1 (YAP1) in Prostate Cancer Cells: A Potential Cross-Talk between PDEF and Hippo Signaling

**DOI:** 10.3390/ph12040181

**Published:** 2019-12-10

**Authors:** Praveen Kumar Jaiswal, Suman Mohajan, Sweaty Koul, Fengtian Wang, Runhua Shi, Hari K. Koul

**Affiliations:** 1 Department of Biochemistry and Molecular Biology, LSU Health Sciences Center Shreveport, LA 71130, USA; pjaisw@lsuhsc.edu (P.K.J.); smohaj@lsuhsc.edu (S.M.); fwang1@lsuhsc.edu (F.W.); 2 Department of Urology, LSU Health Sciences Center Shreveport, LA 71130, USA; skoul@lsuhsc.edu; 3 Feist Weiller Cancer Center, LSU Health Sciences Center Shreveport, LA 71130, USA; RShi@lsuhsc.edu

**Keywords:** YAP1, PDEF, prostate cancer

## Abstract

PDEF (prostate-derived ETS factor, also known as SAM-pointed domain containing ETS transcription factor (SPDEF)) is expressed in luminal epithelial cells of the prostate gland and associates with luminal phenotype. The Hippo pathway regulates cell growth/proliferation, cellular homeostasis, and organ development by modulating phosphorylation of its downstream effectors. In previous studies, we observed decreased levels of PDEF during prostate cancer progression. In the present study, we evaluated the effects of the expression of PDEF on total/phosphoprotein levels of YAP1 (a downstream effector of the Hippo pathway). We observed that the PC3 and DU145 cells transfected with PDEF (PDEF-PC3 and PDEF-DU145) showed an increased phospho-YAP1 (Ser127) and total YAP1 levels as compared to the respective PC3 vector control (VC-PC3) and DU145 vector control cells (VC-DU145). We also observed an increased cytoplasmic YAP1 levels in PDEF-PC3 cells as compared to VC-PC3 cells. Moreover, our gene set enrichment analysis (GSEA) of mRNA expression in PDEF-PC3 and VC-PC3 cells revealed that PDEF resulted in inhibition of YAP1 target genes, directly demonstrating that PDEF plays a critical role in modulating YAP1 activity, and by extension in the regulation of the Hippo pathway. We also observed a decrease in YAP1 mRNA levels in prostate cancer tissues as compared to normal prostate tissues. Our analysis of multiple publicly available clinical cohorts revealed a gradual decrease in YAP1 mRNA expression during prostate cancer progression and metastasis. This decrease was similar to the decrease in PDEF levels, which we had reported earlier, and we observed a direct correlation between PDEF and YAP1 expression in CRPC data set. To the best of our knowledge, these results provide the first demonstration of inhibiting YAP1 activity by PDEF in any system and suggest a cross-talk between PDEF and the Hippo signaling pathway.

## 1. Introduction

Prostate cancer (PCa) is the second most common cause of cancer deaths in men in the USA. Despite advancements in the early diagnosis and treatment of localized PCa, about 31,620 men will die of PCa in 2019, mostly due to metastatic castrate-resistant prostate cancer (mCRPC). The progression of PCa initially depends on androgen receptor (AR) signaling. Androgen deprivation therapy (ADT) is the primary treatment option for PCa [1]. However, androgen deprivation therapy fails and leads to the development of castrate resistance prostate cancer (CRPC), which is a continuum of an advanced/aggressive stage of PCa [2]. Patients with the CRPC phenotype are poor responders to available current therapy, including the second-generation drugs, e.g., enzalutamide [3]. 

PCa is associated with dysregulation of many signaling pathways. One of the important signaling pathways that control cell growth/proliferation, cellular homeostasis, and organ development, is the Hippo pathway [4]. This tumor suppressor pathway was first identified in *Drosophila melanogaster* [5] and is highly conserved across species, including humans [6]. The downstream effector of the Hippo pathway is YAP (Yes-associated protein, also known as YAP1). YAP lacks a DNA-binding domain and interacts with other transcription factors, such as Transcriptional Enhanced Associate Domain (TEAD), to bind DNA and regulate gene expression [7]. Multiple signaling events such as cell–cell contact, cell density/polarization, mechano-transduction, G-protein coupled receptor-mediated signaling regulate Hippo pathway activation [8]. 

Altered expression of YAP1 has been associated with many solid tumors, including PCa [9,10,11,12,13,14,15,16,17,18]. The role of PDEF (prostate-derived ETS factor, also known as SAM-pointed domain containing ETS transcription factor (SPDEF)) in PCa remains highly debated [19,20,21,22,23,24,25,26]. We observed that PDEF expression is decreased during PCa progression and that PDEF suppresses the epithelial–mesenchymal transition (EMT) and metastasis in part by driving the expression of epithelial/luminal differentiation-related genes [21,25]. Present studies investigated the relationship between PDEF expression and YAP1 activity, a readout of the Hippo signaling pathway, in PCa.

We observed that the expression of PDEF in PC3 cells resulted in increased levels of YAP1 and phospho-YAP1 (Ser127) protein, increased phospho-YAP1 (Ser127)/total YAP1 ratio, and a negative enrichment of YAP1 conserved signature. We also observed a gradual decrease in YAP1 mRNA expression during prostate cancer progression (low to high Gleason grade and during metastasis). Analysis of YAP1 and PDEF in the neuroendocrine prostate cancer (NEPC)/CRPC dataset showed a further decrease in YAP1 as well as PDEF mRNA levels in NEPC as compared to CRPC, and a direct correlation between PDEF and YAP1 expression. These exciting results show for the first time the inhibition of YAP1 transcriptional activity by PDEF, and a potential cross-talk between PDEF and the Hippo pathway.

## 2. Results

### 2.1. Expression of PDEF in PC3 and DU145 Cells Results in Increased YAP1 and Phospho-YAP1 Protein (Ser127), An Increased Phospho-YAP1 Protein (Ser127)/YAP1 Protein Ratio, and Negative Enrichment of YAP1 Target Genes

**Figure 1:**
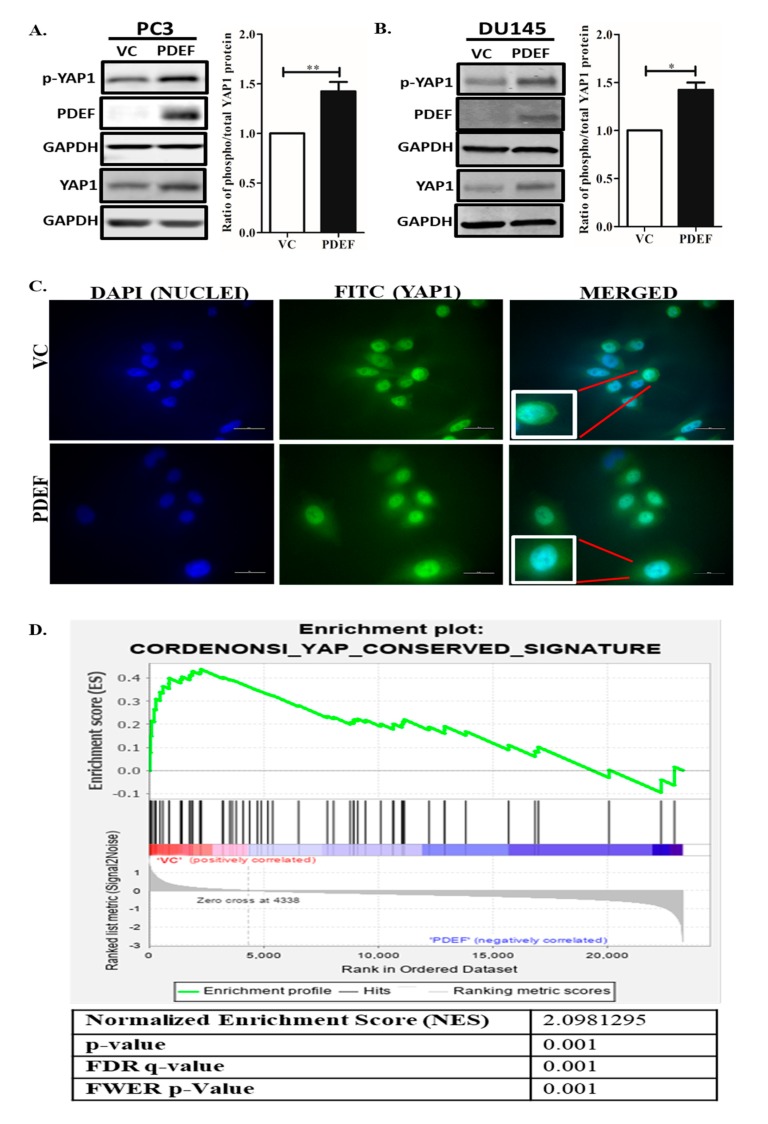
Effect of prostate-derived ETS factor (PDEF) on YAP1 and Phospho-YAP1 Protein (Ser127) protein levels and YAP1 transcriptional activity in prostate cancer cells in culture. (**A**) PDEF-PC3 cells have a higher amount of phospho-YAP1 protein and total YAP1 protein as compared to VC-PC3 cells. (**B**) PDEF-DU145 cells have a higher amount of phospho-YAP1 protein and total YAP1 protein as compared to VC-DU145 cells. The adjacent graph is quantitation of western blots ratio of phospho (Ser127)/phospho/total YAP1 protein level. (**C**) PDEF-PC3 cells showed more cytoplasmic distribution of YAP1 protein, while VC-PC3 cells showed more nuclear localization of YAP1 protein (scale bar 20 µm). (**D**) Gene set enrichment analysis (GSEA) of the YAP conserved gene signature in VC-PC3 and PDEF-PC3 cells.

To investigate the relationship between PDEF and YAP1, levels of total YAP1 and phosphorylated YAP1 (Ser127) protein were analyzed in PDEF-PC3 and PDEF-DU145 cells [21], and VC-PC3/VC-DU145 cells by western blots. We observed that PDEF-PC3 and PDEF-DU145 cells have a higher amount of YAP1 protein levels (total and phosphoprotein (Ser127) levels) as compared to VC-PC3/VC-DU145 cells (Figure 1A,B). Moreover, quantitation of Phospho-YAP1 Protein (Ser127) and YAP1 protein levels revealed that PDEF expression results in an increased Phospho-YAP1 Protein (Ser127)/YAP1 protein ratio, suggesting potential inhibition of YAP1 mediated transcription. Furthermore, analysis by immunofluorescence (IMF) for YAP1 showed more cytoplasmic YAP1 levels in PDEF-PC3 cells as compared to VC-PC3 cells (Figure 1C). To further elucidate the mechanistic role of PDEF in regulating YAP1 levels, we analyzed mRNA expression data generated in the Affymetrix format, from PDEF-PC3 and VC-PC3 cells that we have described previously (GSE108641) [25]. Gene set enrichment analysis (GSEA) of mRNA expression in PDEF-PC3 and VC-PC3 cells revealed that PDEF inhibits expression of YAP1 target genes (Figure 1D), directly demonstrating that PDEF plays a critical role in modulating YAP1 transcriptional activity, and by extension in the regulation of the Hippo pathway. These results are the first direct demonstration of regulation of YAP1 by PDEF in any system. 

### 2.2. YAP1 mRNA Expression is Decreased in PCa Patients from Different Clinical Cohorts

**Figure 2:**
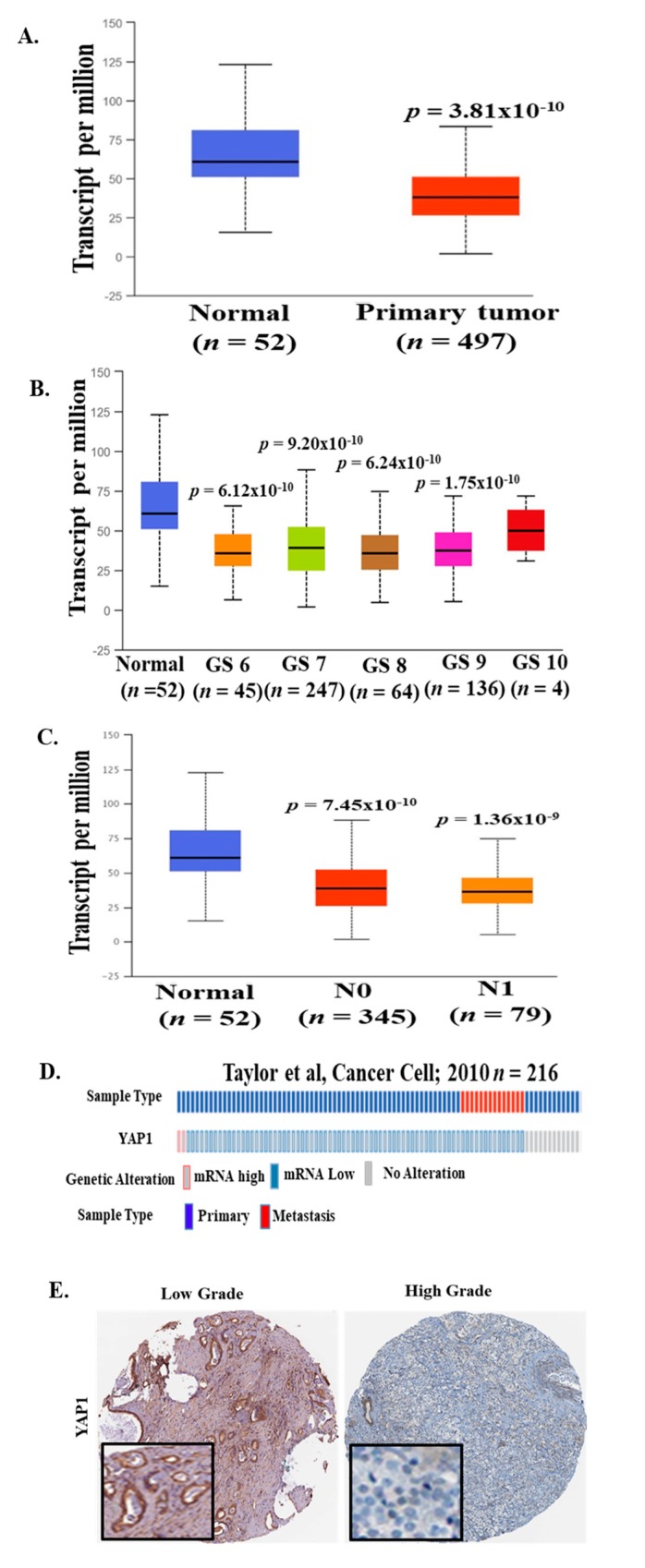
YAP1 mRNA expression in multiple PCa clinical cohorts. YAP1 mRNA data were analyzed from The Cancer Genome Atlas (TCGA) datasets through UALCAN and the c-Bioportal web server. (**A**) mRNA level of YAP1 was significantly decreased in primary prostate tumors as compared to normal prostate tissues. (**B**) A decrease in YAP1 mRNA levels was observed in patients with a higher Gleason score (TCGA datasets) as compared to normal controls. (**C**) YAP1 mRNA levels were significantly decreased in PCa patients irrespective of lymph node metastasis as compared to normal controls (TCGA datasets). (**D**) 51% of patients showed a genetic alteration in YAP1 mRNA levels in the Prostate Adenocarcinoma MSKCC dataset. [31] (**E**) Representative images is from The Human Protein Atlas showed a decreased level of YAP1 protein in high-grade PCa tumor samples as compared to low-grade PCa tumor samples.

We analyzed multiple clinical cohorts of PCa for YAP1 mRNA expression using interactive web resource UALCAN [27] and c-Bioportal [28,29]. The results revealed a significant decrease in YAP1 mRNA levels in patients with PCa (*n* = 497) as compared to the normal control (*n* = 52; *p* = 3.81 × 10^−10^; Figure 2A) in The Cancer Genome Atlas (TCGA) data set [30]. We also analyzed YAP1 mRNA expression data for patients with different Gleason scores (GS). Compared to normal control, we found a significant decrease in YAP1 mRNA levels in all grades of PCa (GS6; *p* = −6.12 × 10^−10^, GS7; *p* = 9.20 × 10^−10^, GS8; *p* = 6.24 × 10^−10^ and GS9; *p* = −1.75 × 10^−10^) (Figure 2B). Moreover, YAP1 mRNA levels were significantly decreased in PCa patients irrespective of lymph node metastasis (N0, *p* = 7.45 × 10^−10^; *n* = 345 and N1, *p* = −1.36 × 10^−9^; *n* = 79) as compared to normal controls (*n* = 52; Figure 2C). Further analysis of YAP1 mRNA data in Prostate Adenocarcinoma MSKCC dataset [31] (*n* = 216) revealed that 51% of patients have decreased YAP1 mRNA levels (Figure 2D). These data suggest that a decrease in YAP1 mRNA expression might be an early event in prostate cancer.

### 2.3. YAP1 Protein Levels in High-Grade PCa

To elucidate the status of YAP1 protein levels in a PCa patient’s sample, we reviewed the immunohistochemistry (IHC) images for YAP1 protein expression across PCa samples in The Human Protein Atlas website [32]. There appears to be a discrepancy with respect to YAP-1 expression, subject to what antibody was used for IHC analysis. A representative image is shown in Figure 2E. These data suggest decreased levels of YAP1 protein in patients with a high-grade PCa tumor as compared to low-grade PCa tumor. These observations are in line with mRNA expression data analyzed from several clinical cohorts.

### 2.4. YAP1 and PDEF mRNA Expression is Lost in NEPC Patients

**Figure 3:**
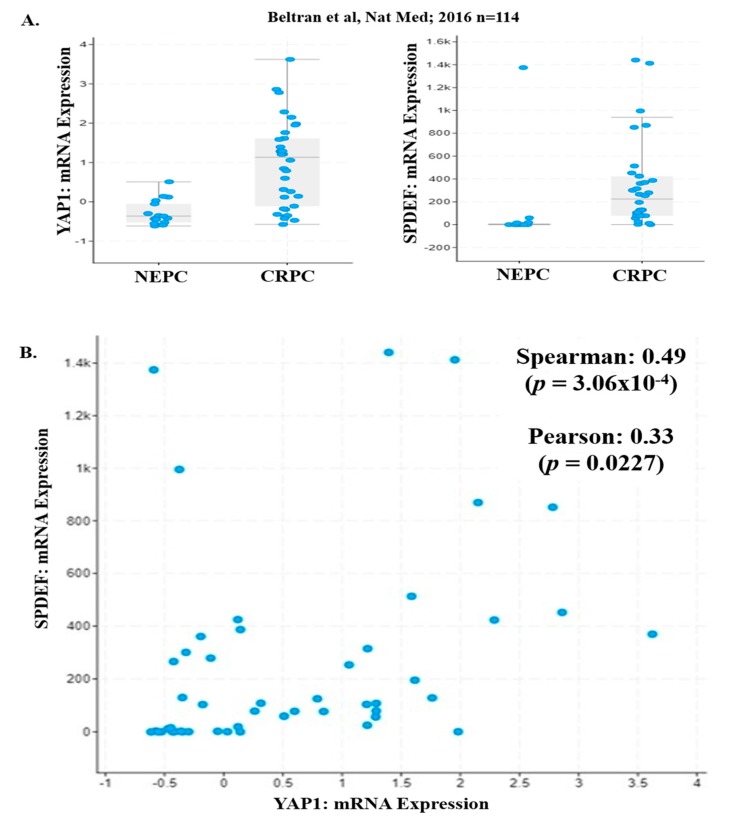
PDEF and YAP1 mRNA levels in CRPC/NEPC patients. (**A**) YAP1 and PDEF mRNA levels were significantly decreased in NEPC patients as compared to CRPC patients (CRPC/NEPC dataset). [33] (**B**) A significant positive correlation was observed between YAP1 and PDEF mRNA in the CRPC/NEPC dataset.

We analyzed the NEPC/CRPC [33] dataset for expression of YAP1 and PDEF mRNA levels. Our analysis of NEPC/CRPC dataset revealed that expression (mRNA levels) of YAP1 and SPDEF was decreased in NEPC patients as compared to CRPC patients (Figure 3A and Table 1). Furthermore, we observed a significant positive correlation (Spearman *p* = 3.065 × 10^−4^, Pearson *p* = 0.0227) between YAP1 and PDEF mRNA with a Spearman coefficient (r) of 0.49 (Figure 3B). These results show for the first time that transition to NEPC is associated with complete loss of PDEF expression and suggests the plausible role of PDEF and YAP1 in the NEPC/CRPC phenotype. 

## 3. Discussion

In the present studies, we observed that the expression of PDEF in prostate cancer cells results in increased levels of YAP1 and phospho-YAP1 (Ser127) protein and an overall increased ratio of phospho-YAP1 (Ser127)/total YAP1. ETS transcription factors have been associated with tumor progression as well as therapy resistance in several cancers, including prostate cancer. However, the role of PDEF in prostate cancer remains debated [19,20,21,22,23,24,25]. Others and we have demonstrated that PDEF limits PCa cell migration, invasion, and clonogenic activity, but the mechanisms by which PDEF regulates these diverse functions are not completely understood. YAP1 is a downstream effector molecule in the Hippo signaling pathway. Hippo signaling modulates cellular functions by regulating the phosphorylation of YAP1; thus, decreased YAP1 expression in prostate cancer cells might render prostate cancer cells resistant to modulation of the Hippo pathway. In light of the above discussion, our observation of increased expression of YAP1 by PDEF in prostate cancer cells suggests that PDEF by regulating expression of YAP1 could sensitize prostate cancer cells to modulation by the Hippo pathway. Indeed, we also observed increased phospho (Ser127)-YAP1 following PDEF expression demonstrating re-establishment of an active Hippo signaling cascade. However, the mechanism by which PDEF regulates YAP1 expression/phosphorylation and the consequences of these effects need additional studies. It has been proposed that the activated Hippo signaling pathway activates MST1/2 kinase that activates and phosphorylate LATS1/2 kinase [34,35]. Activated LATS1/2 kinase then phosphorylates YAP at serine 127 that triggers interaction of phospho-YAP with 14-3-3 complex proteins and that leads to cytoplasmic retention and ubiquitin-mediated degradation of YAP protein. Inactivation of the Hippo pathway results in decreased phosphorylation of YAP and its nuclear localization [36,37] and is associated with an increased YAP1 gene signature (YAP1 target genes). Further studies are warranted to understand the molecular mechanisms by which PDEF expression modulates Hippo signaling. 

Several studies have explored the role of YAP1 in PCa and CRPC [14,15,16,17]. The YAP1–AR axis appears to play a role in prostate cancer progression [15]. YAP regulates cell motility, invasion, and castration-resistant growth of prostate cancer cells [17]. While most of the studies reported YAP1 protein levels in PCa samples, the first study to describe YAP1 expression in CRPC with neuroendocrine differentiation revealed YAP1 mRNA downregulation in NEPC patients in their cohort [18]. The results of YAP1 down regulation in NEPC presented in our study are in line with the above study. In addition, our studies revealed for the first time that YAP1 mRNA levels are decreased in PCa as compared to control normal tissue. Thus, there appears to be a discrepancy between mRNA and protein expression data in prostate cancer. The present study investigated the mechanistic relationship between PDEF and its role in YAP1 regulation in PCa for the first time. Interestingly, PDEF is an AR co-activator and it is conceivable that PDEF plays an important role in the AR-mediated YAP1 axis. There are decreased YAP1 mRNA levels in high-grade tumors as compared to low-grade tumor samples, which parallels that of PDEF.

With the advent of next-generation AR-targeted therapies (abiraterone acetate and enzalutamide), there is an increased burden of lethal therapy-resistant, NEPC prostate cancer for which current treatments are ineffective [2,3,33]. There is an unmet need at present for the identification of molecular markers, and that can be exploited for diagnostic and therapeutic intervention in NEPC. Our exciting observations, that PDEF expression is completely lost in clinical specimens of NEPC patients and that this parallels the loss of YAP1 expression, are tempting to speculate the role of PDEF/YAP1 in reversing the NEPC phenotype to luminal phenotype. Interestingly, our previous studies [25] revealed that expression of PDEF in PC3 cells resulted in the re-establishment of the gene expression signature associated with the prostate luminal epithelial phenotype. However, additional studies are warranted to support the reversal of the NEPC phenotype to epithelial/luminal phenotype by reactivation of the Hippo signaling.

**Figure 4:**
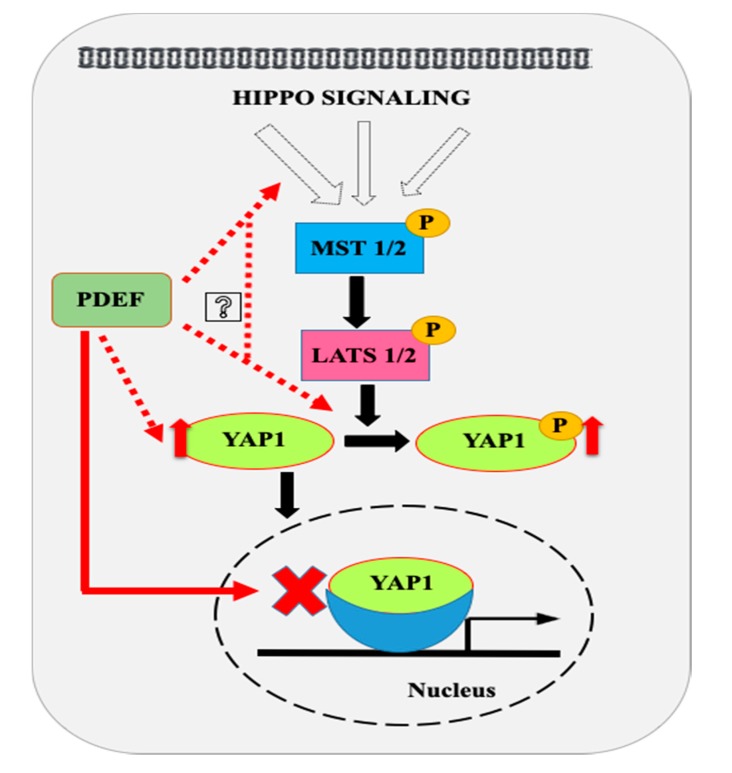
Proposed model for modulations of Hippo pathway by PDEF. Our results show that PDEF increases expression and phosphorylation (Ser127) of YAP1 in prostate cancer cells. We hypothesize that PDEF might regulate YAP1 phosphorylation indirectly by modulating expression and or activities of various components of the Hippo pathway as shown. PDEF overexpression inhibits the YAP1 conserved gene signature. Dashed red lines indicate hypothetical links, while solid black lines indicate currently established pathways. The solid red line link was established for the first time in the present study.

Decreased YAP1 may lead to distorted Hippo signaling and may render advanced prostate cancer impervious to the modulators of the Hippo signaling. Moreover, loss of hippo signaling may result in activation of YAP1 transcriptional program. We found that PDEF-PC3 and PDEF-DU145 cells have higher YAP1 protein levels as compared to VC-PC3/VC-DU145 cells, suggesting that YAP1 levels can be restored by PDEF, which may, as such, re-sensitize the advanced prostate cancers to regulators of the Hippo signaling pathway. This possibility became apparent as we also observed increased phospho-YAP1 (Ser127) levels and increased cytoplasmic ratio in PDEF-PC3 cells as compared to VC-PC3 cells, pointing to the restoration of the Hippo signaling pathway in these cells upon PDEF expression. Finally, we provide direct evidence of inhibition of YAP1 target genes by PDEF. This is significant, as we have demonstrated previously that PDEF promotes epithelial/epithelial luminal phenotype in prostate cancer cells [25]. Based on our results to date, we propose a working model (Figure 4) with respect to the potential mechanism by which PDEF regulates Hippo signaling. Additional studies are warranted to evaluate the effects of such a cross-talk between PDEF and YAP1/Hippo signaling pathway in modulating phenotypic changes in aggressive PCa. 

## 4. Materials and Methods

### 4.1. Materials 

Antibodies against PDEF (Santa Cruz Biotechnology, Inc. sc-166846, 1:1000 dilution), GAPDH (Sigma G8795, 1:3000 dilution), YAP1 (Santa Cruz Biotechnology, Inc. sc-101199, 1:1000 dilution for western blots, 1:50 dilution for Immunofluorescence), and phospho-YAP1 (Cell Signaling Technology (CST) cst-13008, 1:1000 dilution) were purchased from respective vendors.

### 4.2. Cell Lines and Culture 

We used prostate cancer cell line PC3 and DU145 obtained from the American Type Culture Collection (ATCC). PC3 and DU145 cell lines were stably transfected with PDEF and cultured as described previously [25]. 

### 4.3. Data Mining from Multiple Clinical Cohorts

We analyzed the YAP1 mRNA expression data from PCa TCGA [30] (The Cancer Genome Atlas) data sets through UALCAN [27] (http://ualcan.path.uab.edu/) and the c-BioPortal [28,29] (http://www.cbioportal.org) web server. Further, we analyzed YAP1 mRNA expression in the Prostate Adenocarcinoma MSKCC dataset [31]. YAP1 and PDEF mRNA expression data were also analyzed in the NEPC/CRPC dataset [33] through c-BioPortal.

### 4.4. Immunohistochemistry

We procured IHC images for YAP1 protein in PCa patients with low and high-grade tumors from The Human Protein Atlas website [32] (https://www.proteinatlas.org/ENSG00000137693-YAP1/pathology/prostate+cancer#img). 

### 4.5. Western Blot

Western blots for PDEF, YAP1, and Ser127-phospho-YAP1 protein were performed as described [38]. Blots were scanned by a LI-COR Odyssey CLx (LI-COR, Lincoln, USA) system by using IRDye 680 goat anti-mouse/IRDye 800 goat anti-rabbit secondary antibodies as described [39].

### 4.6. Immunofluorescence 

Immunofluorescence for YAP1 protein was done as described [25].

### 4.7. Gene Set Enrichment Analysis (GSEA) 

GSEA was performed using default settings [25], and gene sets were extracted from MysigDB v6.0 (http://software.broadinstitute.org/gsea/msigdb/index.jsp). Details of the analyzed YAP conserved gene signature dataset was retrieved [40].

## 5. Conclusions 

Our findings show for the first time the potential regulatory role of PDEF in activation of the Hippo pathway in prostate cancer. To the best of our knowledge, these results provide the first demonstration of the regulation of the Hippo pathway by PDEF in any system.

## Figures and Tables

**Table 1:** mRNA expression of YAP1 and PDEF in NEPC/CRPC patients.

Gene Name	Median Expression in NEPC	Median Expression in CRPC	*p*-Value
**YAP1**	−0.3623018	1.1336036	3.22 × 10^−5^
**PDEF**	0.4419626	224.12931905	6.47 × 10^−7^
